# Supplementary material for: Enhancing the Cell-Free Expression of Native Membrane Proteins by In Silico Optimization of the Coding Sequence—An Experimental Study of the Human Voltage-Dependent Anion Channel
Source: Membranes (Basel). 2021 Sep 28;11(10):741. doi: 10.3390/membranes11100741 (PMC8540592; doi:10.3390/membranes11100741)
Supplement: Supplementary file 1 [file membranes-11-00741-s001.zip › membranes-1309211-supplementary.pdf]

membranes

MDPI

Supplementary Materials

Enhancing the Cell-Free Expression of Native Membrane Proteins by in Silico Optimization of the Coding Sequence—An Experimental Study of the Human Voltage-Dependent Anion Channel

Sonja Zayni [1], Samar Damiati [2,3], Susana Moreno-Flores [4], Fabian Amman [5,*], Ivo Hofacker [5,6], David Jin [7] and Eva-Kathrin Ehmoser [1,*]

[1] Department of Nanobiotechnology, Institute for Synthetic Bioarchitectures, University of Natural Resources and Life Sciences, Vienna (BOKU), Muthgasse 11, A-1190 Wien, Austria; sonja.zayni@boku.ac.at
[2] Department of Biochemistry, Faculty of Science, King Abdulaziz University, Jeddah 21413, Saudi Arabia; sdamiati@kau.edu.sa
[3] Science for Life Laboratory, Department of Protein Science, Division of Nanobiotechnology, Royal Institute of Technology, 171 21 Stockholm, Sweden; samar.damiati@scilifelab.se
[4] Independent Researcher, 1190 Vienna, Austria; smf8097@gmail.com
[5] Department of Theoretical Chemistry, University of Vienna, Währinger Straße 17, A-1090 Wien, Austria; ivo@tbi.univie.ac.at
[6] Research Group Bioinformatics and Computational Biology, Faculty of Computer Science, University of Vienna, Währinger Straße 29, A-1090 Wien, Austria
[7] Avalon Globocare Corp., 4400 Route 9 South, Suite 3100, Freehold, NJ 07728, USA; david@avalon-globocare.com
* Correspondence: fabian@tbi.univie.ac.at (F.A.); eva.ehmoser@boku.ac.at (E.-K.E.)

Experimental Details

Cloning and Purification of Plasmids–Primer Templates

Primer were purchased from Invitrogen (Thermo Fisher, Waltham, USA). The forward primers are schematically listed in the main text (Figures 1 and 2), and obey the following design:

5′GGGG-attB1-GC-SD-spacer nucleotides-ATG-(downstream box)-3′

where "attB1" is the one-side flanking sequence required for creating the Gateway® entry clone (BP reaction), SD refers to the Shine-Dalgarno sequence forming part of the RBS together with additional nucleotides. Downstream box refers to the nucleotide sequence preceding the protein coding sequence in the translated region (TR). The sequence of the reverse primer, common to all constructs, is the following:

GGGG-attB2-T-TTA-CTCGAGTGCTTGAAATTCCAGTCC

where "attB2" is the second one-side flanking sequence for the Gateway® entry clone.

Table S1. List of primer sequences used in this study. Sequences of untranslated (UTR) and translated (TR) regions are in lowercase and uppercase letters, respectively. UTRs may contain Shine-Dalgarno (red), the Kozak sequence (grey), and spacer sequences (blue and light blue, respectively). The start codon ATG in green. TRs may contain downstream PDEST-17-codons (brown), the CAT enhancer sequence (orange), in addition to the VDAC sequence (black). Single-nucleotide mutations (synonymous mutations) are depicted in pink (Original first-nine-codon sequence: GCT GTG CCA CCC ACG TAT GCC GAC).

Name of Construct	Primer Sequence (5′ to 3′)
VDAC-I-0, II-0	gc gaa gga gat aga acc **ATG** GCT GTG CCA CCC ACG TAT G
VDAC-I-A, II-A	gc gaa gga gat aga **ATG** GCT GTG CCA CCC ACG TAT G
VDAC-I-B, II-B	gc **ATG** GCT GTG CCA CCC ACG TAT G
VDAC-II-C	gc gaa gga gat ata cat **ATG** GCT GTG CCA CCC ACG TAT G

VDAC-II-D	gc gaa gga gat ata cat **ATG** TCG TAC TAC GCT GTG CCA CCC ACG TAT G
VDAC-II-E	gc gaa gga gat ata cat **ATG** GAG AAA AAA ATC GCT GTG CCA CCC ACG TAT G
VDAC-I-C, II-F	gc gaa gga gat aga **ATG** GAG AAA AAA ATC GCT GTG CCA CCC ACG TAT G
VDAC-II-G	gc gaa gga gat aga **ATG** GCA GTA CCA CCA ACA TAC GCA GAC

Cloning and purification of plasmids–PCR cycle program for the amplification of the VDAC-encoding gene.

Denaturation: 98 °C, 10 s ; Annealing: 56 °C, 10 s; Elongation: 72 °C, 20 s.

RNA detection and quantitative PCR (qPCR)–RNA isolation, qPCR experimental conditions.

RNA isolation was carried out with 35 µL of the cell-free reaction mixture and the Aurum Total RNA Mini Kit (Bio-Rad, Hercules, USA), following the protocol for bacterial cells with the exception of adding lysozyme. The isolation procedure includes a DNA digestion step and elution was performed with 40 µL elution buffer before spectrophotometric measurements and reverse transcription into cDNA for qPCR.

qPCR experiments were performed with cDNA at a starting concentration of 500 nM, and three VDAC-specific primer pairs for amplicons of varying lengths, as the table below shows:

Table S2. qPCR VDAC specific primer pairs.

Primer Pair	Forward Primer	Reverse Primer	Amplicon Length (bp)
1	CCAAGGGCTATGGATTTGG	TACTTGGTTTCCAGACTGCC	130
2	ACACTAGGCACCGAGATTAC	GCTTGTACCCTGTCTTGATT	120
3	ACGTGGACTGAAGCTGAC	GCTTGTACCCTGTCTTGATT	80

The PCR reaction mixture was subjected to the following multi-step treatment: 95°C for 180 s (step 1), 95°C for 10 s (step 2); 60°C for 30 s (step 3); 95°C for 10 s (step 4); temperature sweep from 65 to 95°C (melt curve with 5°C temperature steps every 5 s, step 5). Steps 2 and 3 were repeated 40 times, plate reading was done after steps 3 and 5. Lid temperature was 100°C. Sample with primers but no cDNA was used as non-template control (NTC).

Matrix-Assisted Laser Desorption Ionization–Time-of-Flight Mass Spectrometry (MALDI-TOF MS) and MS/MS.

MALDI-TOF MS was conducted on in-gel digested protein expressed from VDAC-I-0. The sample was purified from the cell-free reaction mixture prior to in-gel digestion as described below. The tryptic peptides were isolated, dried, and resuspended in acetonitrile-trifluoroacetic acid before being analysed in an Autoflex MALDI TOF/TOF mass spectrometer (Bruker, Billerica, USA). Peptide matching was performed by comparing the experimental tryptic profile with those from the theoretical profile of VDAC. VDAC-based peptide sequences and comparison were carried out using the MS-Digest and MS-Tag algorithms of *ProteinProspector* (*http://prospector.ucsf.edu*), respectively. Peptide fragments giving out matching peaks were chosen as parent ions for partial sequencing via MS/MS.

MALDI-TOF MS and MS/MS–Protein purification, sample preparation.

HIS-Tagged VDAC was purified using the MagneHis™ Protein Purification Kit (Promega) according to manufacturer's instructions, and eluted from nickel beads in two

fractions. Attempts to filter the eluted fractions with Amicon ultra-0.5 mL centrifugal filters (Millipore) or D-tubeTM dialyzer Mini (MWCO 12–14 kDa, Novagen) led to considerable loss of sample, and hence fractions were not filtered after elution. The first fraction eluted by 3 M NaCl and the second by 3 M NaCl, 500 mM imidazole in 10 mM HEPES (1:1). The second fraction, of higher purity, was used for MS analysis.

In-gel digestion of HIS-tagged VDAC protein for MS analysis was performed as follows. After excising the VDAC band, the gel fragments were distained and dehydrated thrice at room temperature for 15 min, twice with 50% acetonitrile (AcCN) and once with 100% AcCN, before being further treated with 0.1 M NH_4HCO_3 and 100 % AcCN. Gel bands were then dried in a Speed Vac (Thermo Scientific) and then reduced and allowed to swell with 10 mM dithiothreitol (DTT) in 0.1 M NH_4HCO_3 at room temperature for 5 min. The swollen pieces were incubated at 56°C for 45 min, and alkylated with 55 mM iodoacetamide in 0.1 M NH_4HCO_3 in the dark for another 30 min. Gel bands were then washed as detailed above, and dried in AcCN before trypsin-digested in 0.1 M NH_4HCO_3. Trypsin cleaves at the carboxyl sites of positively-charged Lys and Arg side chains. After digestion, the pieces were reswollen for 5 min, then incubated overnight in 0.1 M NH_4HCO_3 at 37°C and the supernatant transferred into a clean tube. Peptide traces still lodged in the gel were extracted thrice with AcCN: H_2O: trifluoroacetic acid (TFA) (660:330:1), incubated at room temperature for 15 min and at 1400 rpm, and added to the tube. The extracted peptides were then dried in the Speed-Vac at low or zero heat, and resuspended in 5 μL 50% AcCN and 0.1% TFA, before dispensed onto the MALDI-target plate and air dried at room temperature. The masses of minimum and maximum digest fragment masses were found to be 500 (5 amino acids) and 4000 Da, respectively.

Database search was carried out with one missing trypsin cleavage, carbamidomethylation and methionine oxidation. Mass tolerances were 0.1% for precursor and product ions. The variable search parameters were Acetyl (Protein N-term), Gln-pyro-Glu (N term Q), Met-loss (Protein N-term M), Met-loss+Acetyl (Protein N-term M), and Oxidation (M).

In-cell expression of VDAC proteins

VDAC-I-0 and VDAC-II-0 plasmids were transformed into *E. coli* BL21 via heat-shock transformation. Cells were then regenerated by incubation in SOC broth at 37°C for 1 h. Transformed cells were allowed to grow overnight on LB agar plates supplemented with 50 μm/mL ampicillin at 37°C. Single colonies were inoculated in 5 mL LB medium containing ampicillin. After overnight growth, 100 μL of the start culture was transferred to 5 mL of fresh LB medium containing ampicillin, and further incubated at 37°C for 3 h. 5 μL of 1 M isopropyl-β-D-thiogalactopyranosid (iPTG), were then added to the culture to induce over expression of VDAC protein, and further incubated at 37°C for another 3 h. 100 μL of sample were collected every hour and checked for protein expression via SDS-PAGE and Western Blot.

Simulated annealing and construct optimization

The procedure starts with the characterization of the source sequence, the RNA transcript of a reference construct. δ is computed with the equation in the main text, with ΔE_{tRNA} set constant and equal to -1.19 kcal/mol for the AUG start codon. ΔE_{SD} is calculated between a 30-nucleotide-long segment upstream the start codon of the source sequence and and the anti-SD sequence in the 16S-ribosomal RNA (CACCTCCTTA), for the most energetically favourable location of the SD sequence in the source, which is obtained with *RNAduplex*. ΔE_{open} is calculated with the program *RNAup*.

δ is then maximised by allowing single-nucleotide mutations in the TR of source sequences that encode the same amino acid (synonymous). The simulated annealing algorithm randomly swaps codons with synonymous mutations and the resulting sequence is characterized as described above. Mutations are accepted as long as they decrease ΔE_{open}; otherwise they are accepted with a probability *p* that in turn depends on the change in

ΔE_{open} brought about by the mutation, and a virtual temperature *t* that is iteratively decreased from 2 to 0:

$$p \le e^{\left(\Delta E_{open}^{after} - \Delta E_{open}^{before}\right)/t}$$

where ΔE_{open}^{before} and ΔE_{open}^{after} are the opening energies before and after introducing the mutation, respectively. Hence the probability of accepting a detrimental mutation is reduced in the course of the iterative process. This allows to widely explore the energy landscape before fine searching for local energy minima. Finding mutations that are accepted is accomplished by setting 300 iteration steps with up to 25 tries per step.

Calculation of accessibility profiles for single constructs and sets of constructs (ΔE_{open} (i))

For the constructs such as those displayed in Figure 4a and b, ΔE_{open} (i) is calculated using *RNAplfold* with the parameters -W 120 -L 70 -u 30. All coding sequences (4,294) from the K12 strain of *E. Coli*, including the 100 nucleotide long upstream region, were obtained from *microbes.uscs.edu*. The cDNA sequence of all human protein transcripts (57,982 sequences) as well as all protein coding transcripts associated with the GO-term "integral component of the membrane" (GO:0016021, 8,270 sequences with an annotated 5′UTR of 100 nucleotides at least) were obtained from *ensemble_biomart*. The UTRs of these transcripts were replaced with the sequence of the pDEST14 vector to generate the pDEST14-based constructs, and the respective sequences of the CAT enhancer (GAGAAAAAAATC) and the embedded HIS-tag (TCGTACTACCATCACCATCACCATCACCTCGAATCAACAAGTTTGTACAAA AAAGCAGGCTGCGAAGGAGATAGAACC) inserted right after the start codon. For the purpose of visualization, ΔE_{open} (i) data were smoothed by a generalized additive model (Wood, S.N. *J. R. Stat. Soc. B: Statistical Methodology* 2001, 73, 3–36).

Results

MALDI-TOF MS and MS/MS

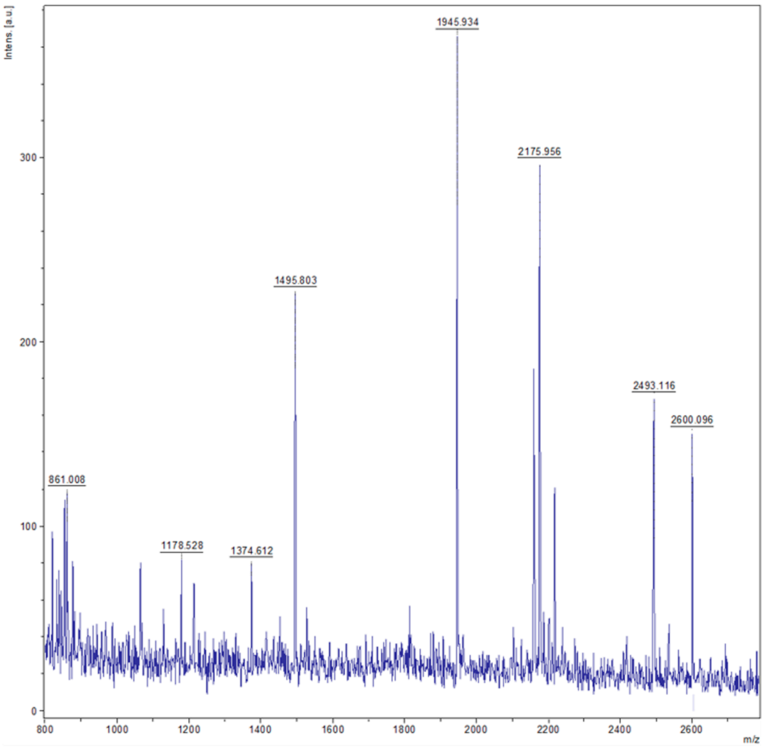

m/z	Intens.	S/N
820,379	93,457	7,406
855,028	91,406	7,266
**861,008**	100,010	7,802
**1178,528**	79,735	6,667
**1374,612**	69,967	6,039
**1495,803**	183,170	15,855
**1945,934**	272,426	25,621
2159,900	127,504	13,877
**2175,956**	243,559	27,139
2217,966	85,606	9,875
**2493,116**	91,691	13,754
**2600,096**	108,255	17,931

1 MAVPPTYADL GKSARDVFTK **GYGFGLIK**LD LKTKSENGLE FTSSGSANTE TTKVTGSLET KYR**WTEYGLT FTEKWNTDNT**

81 **LGTEITVEDQ LAR**GLKLTFD SSFSPNTGKK NAKIKTGYKR **EHINLGCDMD FDIAGPSIR**G ALVLGYEGWL AGYQMNFETA

161 KSRVTQSNFA VGYK**TDEFQL HTNVNDGTEF GGSIYQK**VNK **KLETAVNLAW TAGNSNTR**FG IAAKYQIDPD ACFSAKVNNS

241 SLIGLGYTQT LKPGIKLTLS ALLDGKNVNA GGHKLGLGLE FQALE

Figure S1. MALDI-TOF-MS spectrum of VDAC showing the tryptic peptides. The theoretical masses, intensity and signal-to-noise ratio (S/N) are shown in the box. Peaks corresponding to matching peptide sequences (in red below) are shown in bold type. Below, the complete VDAC amino acid sequence, showing the matching peptide sequences in red and the sites of cleavage by trypsin (_). The matched peptides cover 34% of the protein with 98 identical residues of 285 aminoacids for VDAC.

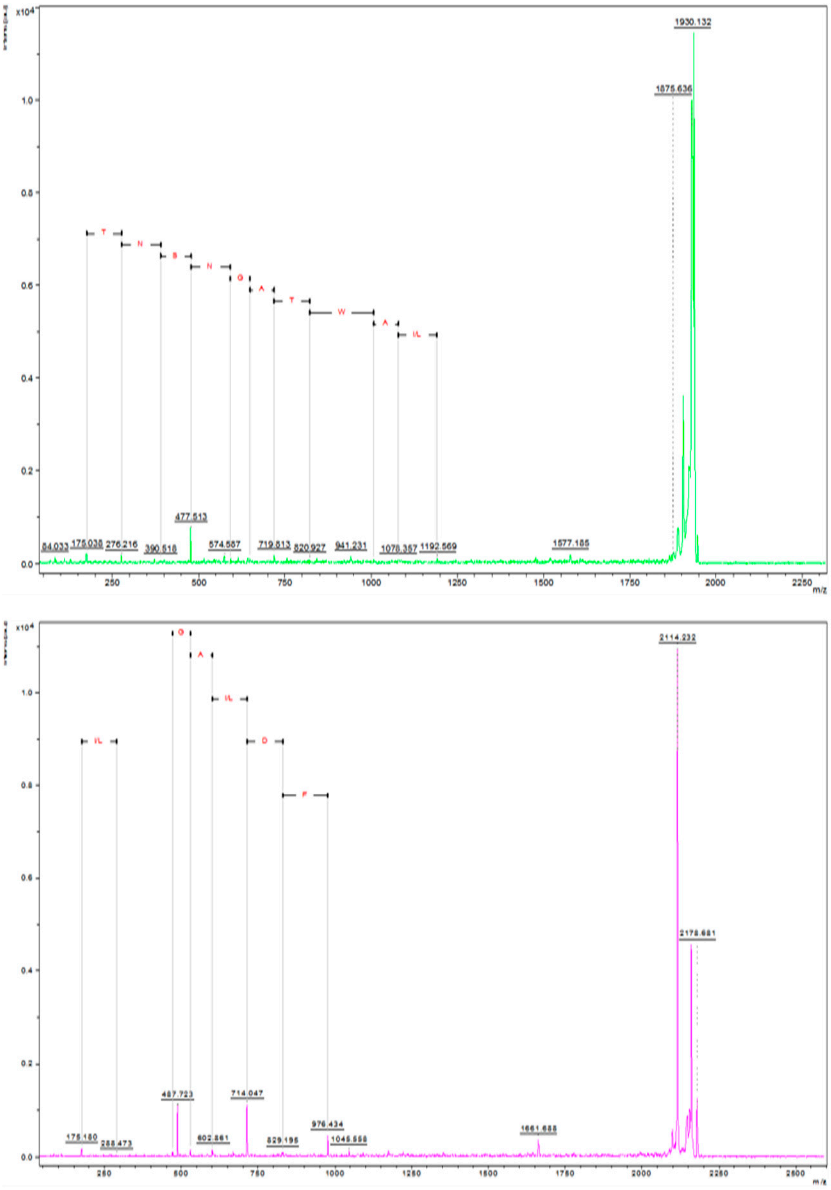

Figure S2. MS/MS spectra of two parent ions corresponding to the two highest peaks of the MS spectrum (figure S1). Upper graph: Spectrum of the peptide 1945.934 m/z with sequence KLETAVNLAWTAGNSNTR (matched intensity: 48.1%). Lower graph: Spectrum of the peptide 2175.956 m/z with sequence EHINLGCDMDFDIAGPSIR (matched intensity: 34.8%).

In-cell expression of VDAC protein

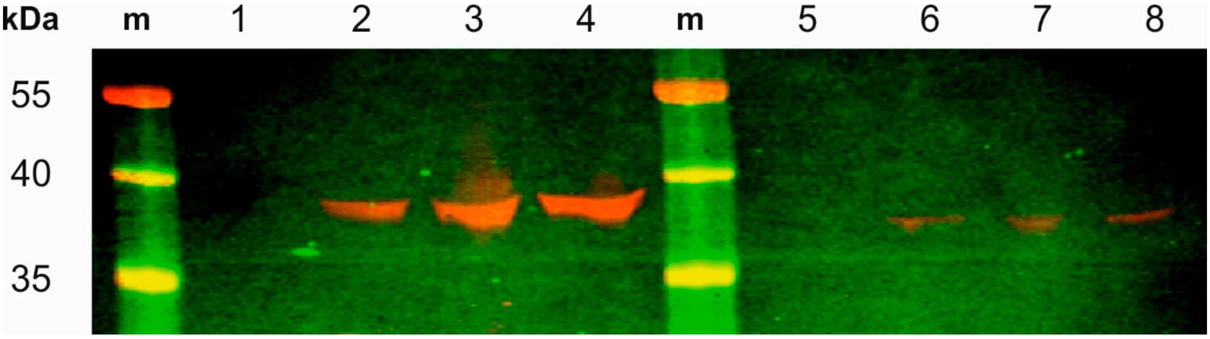

Figure S3. Western Blots of VDAC expression in E. coli. Lanes 1–4: Expression levels using VDAC-I-0 before (1), and 2h (2), 3 h (3), and an overnight (4) after the addition of IPTG. Lanes 5–8: Expression levels using VDAC-II-0 construct before (5), and 2 h (6), 3 h (7), and an overnight (8) after the addition of iPTG.). m stands for marker.

Coomassie and Western Blots–VDAC-I and VDAC-II

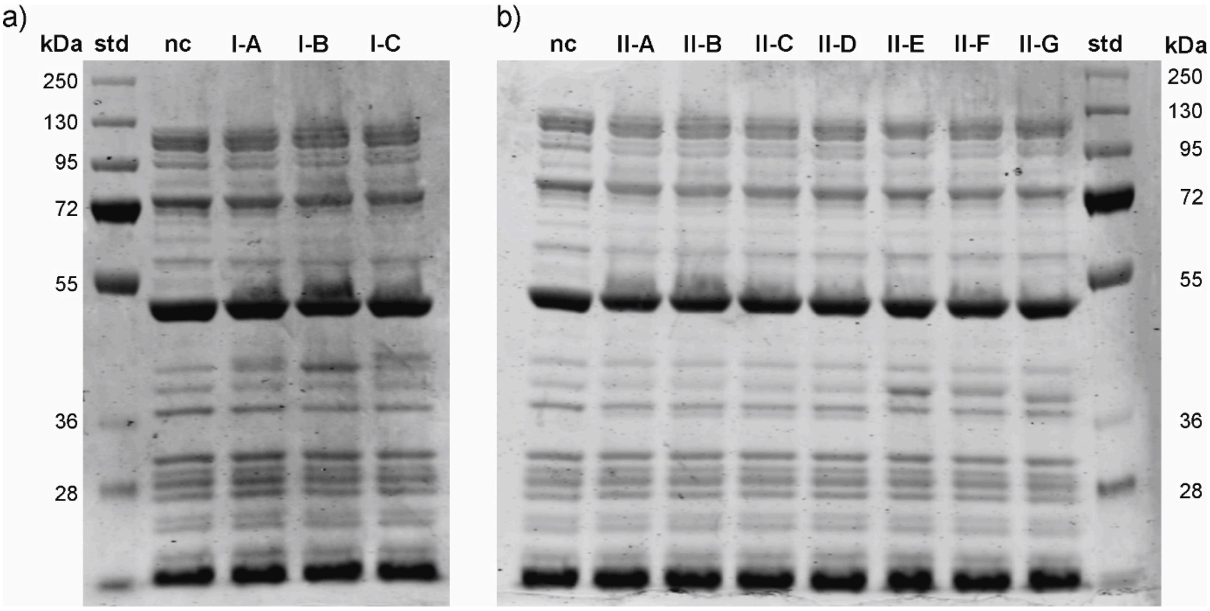

Figure S4. Coomassie stainings of the constructs VDAC-I (A,B,C) (a) and VDAC-II (A-G) (b). Fluorescence scans on the same gel are *Scheme 1.* and 2, respectively. (std: standard; nc: negative control).

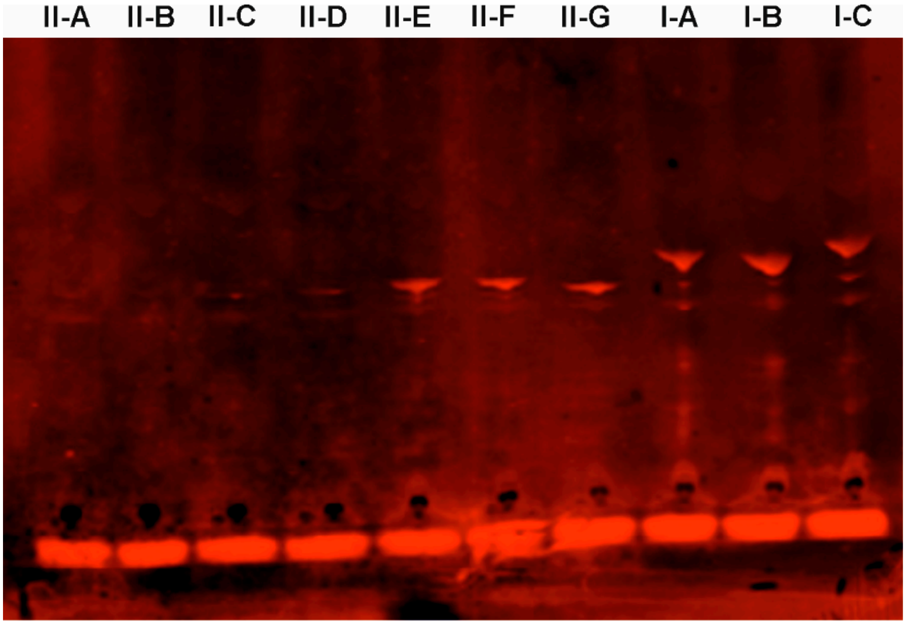

Figure S5. Western Blot showing the level of protein expression for the constructs VDAC-I (I-A, I-B, I-C) and VDAC-II (II-A, II-B, II-C, II-D, II-E, II-F, II-G).

Table S3. Number of amino acids and the corresponding molecular weight (MW) of the resulting protein, based on the construct´s coding sequence.

Construct	Number of Amino Acids of the Coding Protein	Calculated MW of the Coding Protein (kDa)
IA	311	34.12
IB	307	33.66
IC	315	34.12
IIA-IIC	285	31.01
IID	288	31.43
IIE	289	31.51
IIF	289	31.51
IIG	285	31.01

SDS-page and Western Blot–bands at lower Mw

Worth mentioning is the presence in both the SDS-page gel and Western Blot of an additional band at approximately 36 kDa. This band of weak intensity appears only when the intensity of the 39 k Da band is either low or non-detectable; in other words, in the case of plasmids II-A to II-D or of low to no VDAC expression.

Protein expression in the absence of natural or good templates is not unheard of in in-vitro synthesis with bacterial systems, [1] and may be symptomatic of mRNA transcription re-initiation events. Resuming transcription initiation results in shorter mRNA transcripts, and hence to the expression of proteins of lower molecular weight. In this case, the underlying mechanism may not be different from that reported for DNA replication in the presence of non-replicating polymerases, whereby nascent RNA transcripts act as primers to re-initiate DNA replication. [2]

Accessibility profiles ($\Delta E_{open}(i)$)–H. sapiens and E. coli transcripts

Native human mRNA transcripts are less accessible than *E. coli*´s, which is reflected in higher values of ΔE_{open} at the optimal location of the ribosome docking site (RDS), i.e., at *i* = -11 for *E. coli*. The disparity in profiles indicates the existence of structural differences in the translation-initiation region between prokaryotes and eukaryotes.

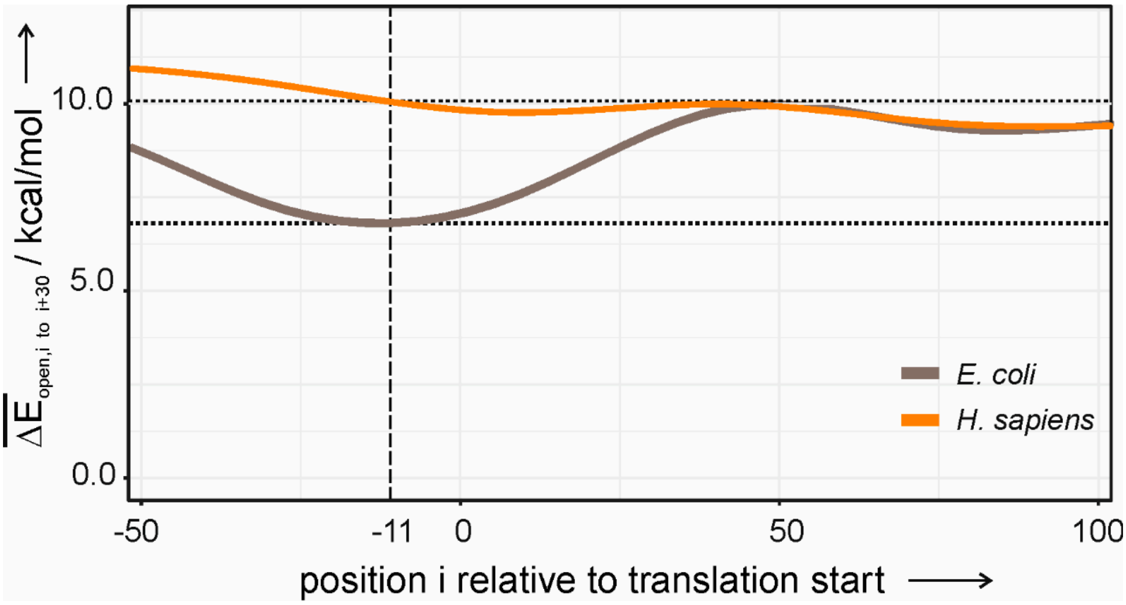

Figure S6. Average opening energies as a function of i, the RDS location for the E. coli (brown curve) and the H. sapiens (orange curve) genomes. Note that at i = -11 the value of ΔE_{open} is a local minimum only in the prokaryotic case.

Reference

[1] A. Arslan Yildiz, *In-vitro synthesis and reconstitution of cytochrome bo3 ubiquinol oxidase in artificial membranes,* PhD Disssertation, **2010.**
[2] S. Bo, A. Singh, S. Sultana, J. T. Inman, S. S. Patel and M. D. Wang, *Nature Comm.,* **2018**, 9, 2306-2314.
